# Development and Optimization of an UPLC-QTOF-MS/MS Method Based on an In-Source Collision Induced Dissociation Approach for Comprehensive Discrimination of Chlorogenic Acids Isomers from *Momordica* Plant Species

**DOI:** 10.1155/2014/650879

**Published:** 2014-09-10

**Authors:** N. E. Madala, F. Tugizimana, P. A. Steenkamp

**Affiliations:** ^1^Department of Biochemistry, University of Johannesburg, P.O. Box 524, Auckland Park 2006, South Africa; ^2^CSIR Biosciences, Natural Products and Agroprocessing Group, Pretoria 0001, South Africa

## Abstract

Chlorogenic acids (CGA) have been profiled in the leaves of *Momordica balsamina*, *Momordica charantia*, and *Momordica foetida*. All three species were found to contain the *trans* and *cis* isomers of 4-acyl *para*-coumaroylquinic acid (*p*CoQA), caffeoylquinic acid (CQA), and feruloylquinic acid (FQA). To the best of our knowledge, this is the first report of *p*CoQA and FQA and their *cis* isomers in these *Momordica* species. These profiles were obtained by a newly developed UPLC-qTOF-MS method based on the in-source collision induced dissociation (ISCID) method optimized to mimic the MS^2^ and MS^3^ fragmentation of an ion trap-based MS. The presence of the *cis* isomers is believed to be due to high UV exposure of these plants. Furthermore, the absence of the 3-acyl and 5-acyl CGA molecules points to a metabolic mark that is unusual and represents a very interesting biochemical phenotype of these species. Our optimized ISCID method was also shown to be able to distinguish between the geometrical isomers of all three forms of CGA, a phenomenon previously deemed impossible with other common mass spectrometry systems used for CGA analyses.

## 1. Introduction

Chlorogenic acids (CGA) are natural antioxidants and one of the most abundant polyphenols in the human diet [1]. These molecules have multiple functions in plants and in most cases; they are produced as a part of the defense mechanism response against environmental stresses triggered by microbial pathogens, mechanical wounding, and direct exposure to UV or visible light [2, 3]. To avoid any ambiguity, in the current study, CGA refer to any molecule which is formed from an ester-bonding between one or more cinnamic acids (*p*-coumaric, caffeic, and ferulic acid) and quinic acid, giving thus rise to* p*-coumaroylquinic acid (*p*CoQA), caffeoylquinic acid (CQA), and feruloylquinic acid (FQA), respectively [2, 3]. In nature, the* trans* isomers are currently known to be more abundant than the* cis* isomers; and the latter are known to accumulate in tissues/cells subjected to intense UV irradiation [4].

From analytical point of view, specialized platforms and approaches are indeed needed to confidently and accurately annotate these molecules, if present in a particular material (e.g., plant). Clifford et al. [5] proposed a mass spectrometry (MS) based method for identification of CGA, underlining the hierarchical fragmentation of these molecules in an MS experiment. Such observed fragmentation patterns pointed to differential behaviors of the CGA isomers during an MS analysis. Even though it has been shown that MS is capable of differentiating the regional isomers, formed from differential positional acylation, this platform is reportedly unable to distinguish between the geometrical isomers (*cis* and* trans*), with the latter resulting in very similar MS-fragmentation patterns [4]. The only way to putatively characterise these geometrical isomers so far is by their differential retention time during a chromatographic separation. In a reverse chromatography, the* cis*-3-acyl and* cis*-4-acyl molecules elute before their* trans* counterparts whilst the opposite is the case for 5-acyl molecule [4].

It is indeed clear that the CGA molecules offer a very interesting analytical challenge that still needs more investigation, chromatographically and at the MS level, for more distinct annotation of different molecular forms of these compounds. In the scientific community, various researchers are therefore spending most of their time designing reliable methods to characterize these molecules (and their associated derivatives) from different sources of plant materials [6]. In the current study, a newly developed UPLC-Q-TOF-MS method, which is based on the in-source collision induced dissociation, is evaluated for the characterization of chlorogenic acid content of the three* Momordica* species growing in the northern part of South Africa.

*Momordica* genus (Cucurbitaceae) is known to have high nutritious values and ethnopharmacological activities [7]. Plants from this genus are known to possess a very bitter taste which has been attributed to the cucurbitacins [8]. These compounds have been shown to exhibit several potent biological activities, namely, cytotoxic, hepatoprotective, anti-inflammatory, cardiovascular, antidiabetic, and antiparasitic [8–10]. To the best of our knowledge, there is very little work done on the other classes of compounds (with potential biological activities) that are possibly present in* Momordica*. In South Africa,* Momordica* species are used as personal prescription medicine to control sugar diabetes. Several studies have indicated that the spectrum of naturally occurring compounds, with biological activities, includes various classes such as phenolics, carotenoids, and flavonoids [11]. CGA have been previously shown to possess antidiabetic properties [12]. Thus, the current study is exploring the presence of chlorogenic acids in* Momordica* found in the northern part of South Africa, with a focus on the Q-TOF-MS-based annotation of the CGA molecules, explaining their fragmentation patterns as a tool to discriminate different forms of CGA. Thus, to achieve the CGA characterization, the in-source collision induced dissociation aided mass spectrometry was used. Partial optimization of the ISCID method for the discrimination of geometrical isomers of CGA is also presented.

## 2. Materials and Methods

### 2.1. Chemicals

All chemicals were of analytical grade quality and were obtained from various international suppliers. Briefly, the organic solvents used were UPLC/MS grade quality methanol (Romil, MicroSep, South Africa) and acetonitrile (Romil, MicroSep, South Africa). Water was purified by a Milli-Q Gradient A10 system (Millipore, Billerica, MA, USA). Leucine enkephalin, 5-caffeoylquinic acid, and formic acid were all purchased from Sigma Aldrich, Germany.

### 2.2. Metabolites Extraction

The leaves of the three* Momordica* species were air-dried using an oven operating at 37°C for three consecutive days. The dried leaves were crushed using a pestle and mortar to a fine to coarse powder with a relative (homogeneous) size. Metabolites were extracted from the crushed leaves (2 g) using 80% MeOH (20 mL). After shaking at room temperature (25°C) for 30 min, the tissue debris was removed by means of centrifugation at 5000 ×g for 10 min. The supernatant was transferred to a new clean tube and dried to at least 1.0 mL using the rotary vapor apparatus at 55°C under vacuum. The 1 mL extract was subsequently dried to completeness using the speed vacuum concentrator centrifuge at 55°C. The resulting pellet was reconstituted in 1.0 mL of 50% aqueous MeOH and filtered through 0.22 *µ*m nylon filters. The filtered 1.0 mL extract was then analysed on an UPLC-MS system.

### 2.3. UPLC-MS Analyses

Chromatographic separation (of the 1 mL extract) was performed on an ACQUITY UPLC system (Waters Corporations, Milford, MA) using a conditioned autosampler at 4°C. One (1)  *µ*L of the extracts was separated on a Waters BEH C8 column (150 mm × 2.1 mm, 1.7 *µ*m), thermostatted at 60°C. A binary solvent mixture was used consisting of water (Eluent A) containing 10 mM formic acid (natural pH of 2.3) and acetonitrile (Eluent B) containing 10 mM formic acid. The initial conditions were 98% A at a flow rate of 0.4 mL/min and were maintained for 1 minute, followed by multiple gradients to 5% A at 26 minutes. The conditions were kept constant for 1 minute and then changed to the initial conditions. The total chromatographic run time was 30 minutes. High definition mass spectrometry was performed on a Waters SYNAPT G1 Q-TOF system in V-optics and operated in electrospray negative mode to enable detection of phenolic compounds. Leucine enkephalin (50 pg/mL) was used as reference lock mass calibrant to obtain typical mass accuracies between 1 and 5 mDa. The optimal conditions of analysis were as follows: capillary voltage of 2.5 kV, the sampling cone at 30 V, and the extraction cone at 4 V. The scan time was 0.1 seconds covering the 100 to 1000 Dalton mass range. The source temperature was 120°C and the desolvation temperature was set at 450°C. Nitrogen was used as the nebulisation gas at a flow rate of 700 L/h.

For targeted approach, the CGA were profiled by collecting MS/MS (typical MS^2^) data of the masses of interest, for CQA at* m/z* 353,* p*CoQA at* m/z* 337, and FQA at* m/z* 367. To achieve the diagnostic fragmentation patterns reported by Clifford et al. [5] of these compounds, the trap collision energy (3–60 eV) and the cone voltage (10–100 V) were experimentally optimised until a stable fragmentation pattern was achieved characterized by the formation of the following stable ions: Q_1_[Quinic acid-H]^−^ at* m/z* 191, C_1_[Caffeic acid-H]^−^ at* m/z* 179, Q_2_[Quinic acid-H-H_2_O]^−^ at* m/z* 173, and C_2_[Caffeic acid-H-CO_2_]^−^ at* m/z* 135. The exact masses,* m/z*, (and their respective elemental formulae) for some of these ions detected in the current study are indicated in Table 1. Most importantly, the ISCID method was optimized to result in a more stable* m/z* 191 (Q_1_) in order to generate this ion upfront inside the source because it was noted, from our preliminary results, that it was ubiquitous in all regional and geometrical isomers. All the acquisition and analysis of data were controlled by Waters MassLynx v4.1 software (SCN 704).

## 3. Results and Discussion

Before embarking on the targeted analysis for profiling chlorogenic acids in the above-mentioned three* Momordica* species (the focus of this study), the UPLC-MS-based untargeted analyses of methanol extracts of the three species revealed inherent differential metabolite content as indicated by principal component analysis (PCA) models of the data (results not shown). This different clustering in PCA models points, thus, to an imminent need for more investigation in the metabolite content of these three species. This untargeted analyses indicated putatively the presence of chlorogenic acids in the methanol extracts of the three* Momordica* species under study.

Though chlorogenic acid molecules are naturally found in most plants, their accurate identification is often very challenging analytically due to the chemical structural forms (in terms of regional and geometrical isomerism) of these compounds. Clifford et al. [5] have developed a more reliable and validated ion trap (IT)-MS-based method for annotation of CGA molecules, exploring hierarchical fragmentation of a molecule in multistage mass spectrometry (MS^*n*^) analyses. The latter are currently possible only on an ion trap MS system. However, with the increased usage of Q-TOF instruments in plant metabolomics (and natural products) research [13–15], it is imperatively important to develop and optimize a Q-TOF based method (for the identification of CGA molecules from plant extracts) which gives accurate results similar to those achieved by an IT-MS platform.

**Figure 1:**
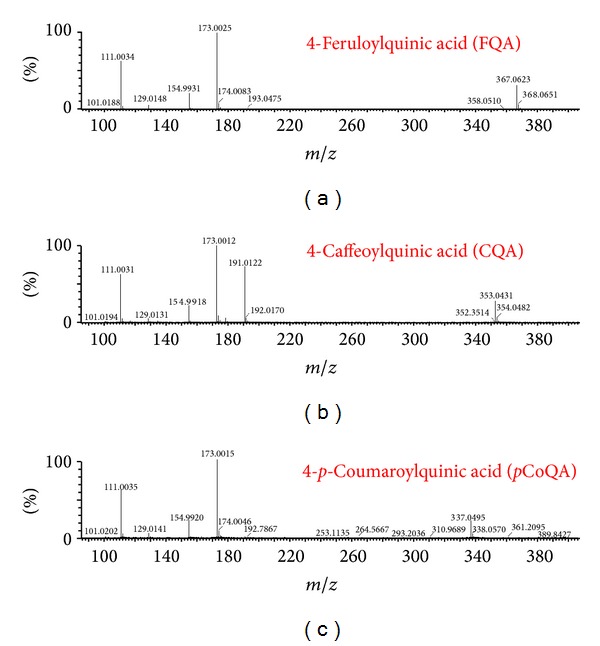
Mass spectra showing the fragmentation patterns of FQA (a), CQA (b) and* p*CoQA (c).

**Figure 2:**
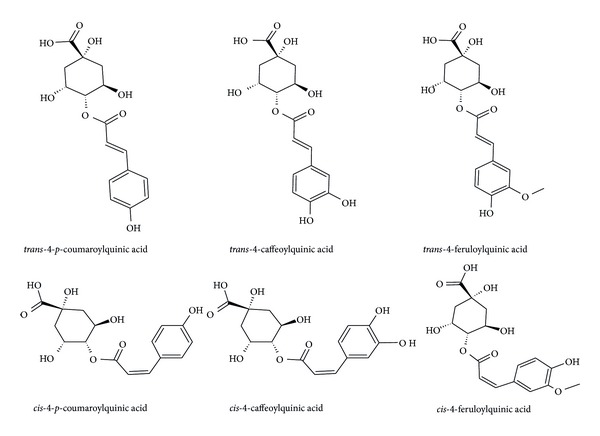
The structures of chlorogenic acids detected in the* Momordica* species.

Thus, to illustrate in more detail our Q-TOF based method for the annotation of CGA molecules, it suffices to highlight some aspects regarding the consistently observed fragmentation patterns of these compounds in MS analyses (MS tuned as detailed in Section 2.2). For instance, in regard to the annotation of CQA molecules, the 4-acylated CQA was identified by the presence of a base peak at* m/z* 173 (Q_2_) (Figure 1, Table 1), which is in agreement with the results generated from an ion trap-MS-based method [5]. The analyses indicated that the 3- and 5-CQA molecules are not present in the* Momordica* species studied. Normally, the MS fragmentation patterns of the 3- and 5-CQA molecules show* m/z* 191 as the base peak for these molecules [5], which is different from the 4-acylated CQA that has the base peak at* m/z* 173 (identified in this study: Figure 1, Table 1). Furthermore, the 3-CQA is distinguished from the 5-CGA by the presence of a base peak at* m/z* 191 with another secondary peak at* m/z* 179 whilst the 5-CQA only shows base peak at* m/z* 191 without a secondary peak at* m/z* 179 [5]. Using a similar approach, other CGA molecules that were confidently annotated in this study are 4-*p*CoQA and 4-FQA molecules (Figure 2). All these observations agree with the ion trap MS results [5].

**Figure 3:**
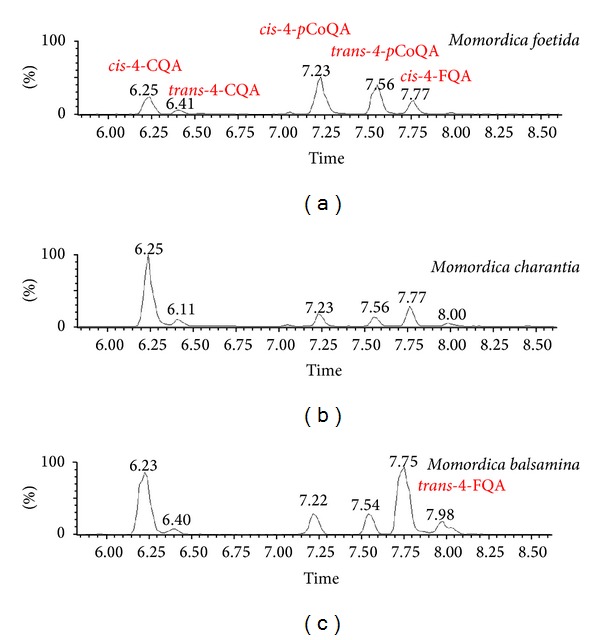
Representative UPLC-Q-TOF-MS BPI chromatograms showing the differential separation of the chlorogenic acids isomers detected in* Momordica* species.

**Figure 4:**
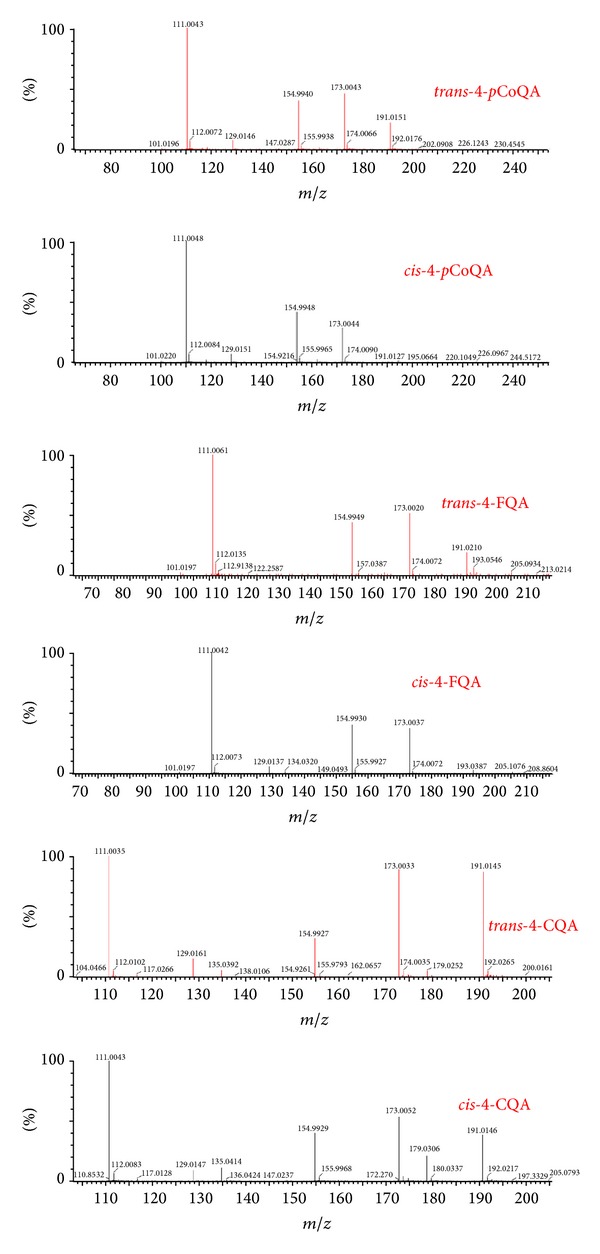
Representative mass spectra showing the major fragmentation differences at* m/z* 191 noted between* trans* and* cis* isomers of all CGA detected in* Momordica* species. The exact mass of* m/z* 191 and its proposed elemental formula are indicated in Table 1.

All the annotated CGA molecules in this study, that is, 4-CQA, 4-*p*CoQA, and 4-FQA, show two ion peaks in their chromatographic separation (Figure 3), which is an indication for the presence of geometrical isomers (*trans* and* cis*). To date, the ion trap-MS-based method has shown that the fragmentation patterns of both* cis* and* trans* chlorogenic acid molecules are very similar, hence the two could not be distinguished by MS analyses [4]. However, in this study, the MS/MS data of* p*CoQA, CQA, and FQA, from our ISCID based method, revealed some clear differences between the* trans* and* cis* isomers of 4-CQA molecule. Here, both the* cis* and* trans* isomers have the base peak at* m/z* 173 [quinic acid-H-H_2_O] (C_1_) and a secondary fragment at* m/z* 191 (Q_1_); however, in all cases, the intensity of the* m/z* 191 fragment (Q_1_) is higher in the* trans* than in the* cis* isomer (Figure 4). It can be postulated that this might be due to energy distribution differences, on quantum level, which is causing these molecules/isomers to behave differently under similar MS conditions. This could be MS-fragmentation signatures that would aid in distinguishing these two geometrical isomers of the 4-CQA molecule.

Our on-going research, as seen from this study (Figure 4), shows that Q-TOF-MS has an extra ability of differentiating geometrical isomers. Furthermore, we note that the obtained fragmentation patterns of CGA molecules from the* Momordica* species (Figures 1 and 4) are in agreement with those of already published data generated from ion trap MS systems (with no MS-based differentiation of* cis* and* trans* isomers) [5, 16] and were confirmed using MS fragmentation of CGA from alcoholic cider and coffee extracts (considered as CGA standards for their known CGA content) which were analysed contemporaneously.

As previously stated, in an attempt to distinguish the geometrical isomers of CGA, Clifford et al. [4] concluded that ion trap MS shows similar fragmentation patterns between* trans* and* cis* isomers. To overcome this inability of IT based MS, Clifford et al. [4] opted for chromatographic retention parameters of these molecules, observing that the* cis* isomers are capable of forming intramolecular hydrogen bonding which is minimal/absent in* trans* isomers. Based on their finding, it was postulated that, in reversed phase chromatography, the* cis* isomers for 3-acyl and 4-acyl elute before their* trans* counterparts and the opposite was observed in the case for 5-acyls. To date, this is the basis in which CGA geometrical isomers are annotated: difference in elution times in reversed phase chromatography.

Here, we suggest thus an MS-based method that differentiates CGA geometrical isomers (*cis* and* trans*) (Figure 4). Similar experiments were conducted in three more CGA sources, namely, the* Moringa oleifera* leaves methanolic extracts,* Nicotiana tabacum* cell cultures methanolic extracts, and alcoholic ciders, resulting substantially in the same observation: differentiation of* cis* and* trans* CGA molecules based on their behavior in the MS. Such an MS-based method of differentiating geometrical isomers of CGA molecules provides an analytical confidence in annotating different forms of CGA molecules (in complex extracts) and is of analytical importance in case of the absence of chromatographic separation.

Even though Q-TOF instrument has been applied for the analyses of chlorogenic acids in plants in the past [13, 17], this platform provided simply a quick insight into possible CGA molecules present in the plant extracts (under study), but with less accuracy and mostly with no distinction among structural variations of the CGA molecules. This limitation of a Q-TOF system is due to its inability to trap ions in step wise process, a trademark of an IT-MS, for multistage MS^*n*^ analyses. Despite such a limitation, the current study develops and suggests a Q-TOF MS approach that could accurately annotate CGA molecules (both regional and geometrical isomers), providing also a particular interpretation of the spectrum from MS fragmentation of these compounds. Furthermore, we report, for the first time, the chlorogenic acid content (qualitatively) of* M. balsamina*,* M. charantia*, and* M. foetida*. The results indicate that all three species contain all forms of the common cinnamic acids (caffeic,* p*-coumaric, and ferulic acid); however, only 4-acylated quinic acids were detected: 4-*p*CoQA, 4-CQA, and 4-FQA (Figures 2 and 3). Further investigations on the ability of Q-TOF-MS to accurately annotate different forms of CGA molecules, distinguishing also their regional/geometrical isomers, are being carried out.

## 4. Conclusion

*Momordica* species, investigated in this study, contain a range of chlorogenic acids, particularly the 4-caffeoylquinic acid, 4-*p*-coumaroylquinic acid, and 4-feruroylquinic acid. However, the absence of other regional isomers (3- and 5-acyls) was unexpected, as they are the most frequently existing forms in many plants. The presence of chlorogenic acids in* Momordica* species, as demonstrated in this study, warrants further investigation by looking at their roles as possible antidiabetic compounds functioning synergistically with the previously identified cucurbitacins. Thus, this study clearly shows the ability of Q-TOF-MS as a technique for metabolite annotation in a mixture of compounds, with a unique promise to distinguish between geometrical isomers (particularly in the case of CGA molecules, as indicated in this study). Thus, fine-tuning the MS settings will definitely result in clearly distinguishable fragmentation patterns between the other chlorogenic acids geometrical isomers (*cis* and* trans*), a phenomenon which has been deemed impossible with ion trap-based MS instruments.

## Figures and Tables

**Table 1:** The UPLC-QTOF-MS/MS information of the annotated CQA, *p*CoQA, and FQA from *Momordica* plants extracts. The diagnostic ions (Q_1_ and Q_2_) used for isomers differentiation are also shown and they were ubiquitous across all the molecules detected.

Rt (min)	[M-H]^−^ Molecular formula	*m*/*z* Measured	*m*/*z* Calculated	Identity
6.25	C_16_H_17_O_9_	353.0873	353.0890	*cis-*4-CQA
6.40		353.0883	353.0873	*trans*-4-CQA
7.23	C_16_H_17_O_8_	337.0864	337.0923	*cis*-4-*p*CoQA
7.56		337.0923	337.0956	*trans*-4-*p*CoQA
7.77	C_17_H_19_O_9_	367.0947	367.1029	*cis*-4-FQA
7.98		367.0933	367.1021	*trans*-4-FQA

Diagnostic ions				
—	C_7_H_11_O_6_ (Q_1_)	191.0473	191.0556	[Quinic acid-H]^−^
—	C_7_H_9_O_5_ (Q_2_)	173.0370	173.0450	[Quinic acid-H-H_2_O]^−^
